# Plant Derived Immunomodulators; A Critical Review

**DOI:** 10.34172/apb.2022.074

**Published:** 2021-10-02

**Authors:** Akey Krishna Swaroop, Chaitanya Motamarri Venkata Naga Lalitha, Meena Shanmugam, Gomathy Subramanian, Jawahar Natarajan, Jubie Selvaraj

**Affiliations:** ^1^Department of Pharmaceutical Chemistry, JSS College of Pharmacy, JSS Academy of Higher Education &Research Ooty, Nilgiris, Tamilnadu, India.; ^2^Department of Pharmacognosy, Chitkara College of pharmacy, Chitkara University, Punjab, India.; ^3^Pharmacy College, Female Sector, Shaqra University, Al Dawadmi, KSA-17431.; ^4^Department of Pharmaceutics, JSS College of Pharmacy, JSS Academy of Higher Education & Research Ooty, Nilgiris, Tamilnadu, India.

**Keywords:** Immunomodulators, Immunostimulants, Immunosuppressants, Traditional plants

## Abstract

The concept of immunomodulation was proposed by Edward Jenner, while working on polio vaccine in 1796. Many of the autoimmune diseases such as rheumatoid arthritis, inflammatory bowel diseases, psoriatic arthritis and system lupus erythematosus, viral diseases and, some cancers are characterized with elevated levels of "immunocytokine" gene expression, including, tumor necrosis factor-α, various interleukins, cytotoxic T-cell antigen-4, B-cell activating factor. For the treatment of these diseases, the immunologically-based therapies play the major role. In these lines, the usage of phytomedicines as immunostimulants/ immunosuppressants have been enhanced considerably in last few decades and also used as a prophylactic treatment for various ailments. Phytochemicals such as flavonoids, terpenoids, polysaccharides, lactones, alkaloids, glycosides and saponins present in several plants, have been confirmed to exhibit immunomodulating properties. This review focuses on the traditional plants and their constituents which have been extensively used as immunomodulators. We have also highlighted the mechanism of action of these plant constituents related to autophagy and adjuvanticity of drugs.

## Introduction

**Figure 1:**
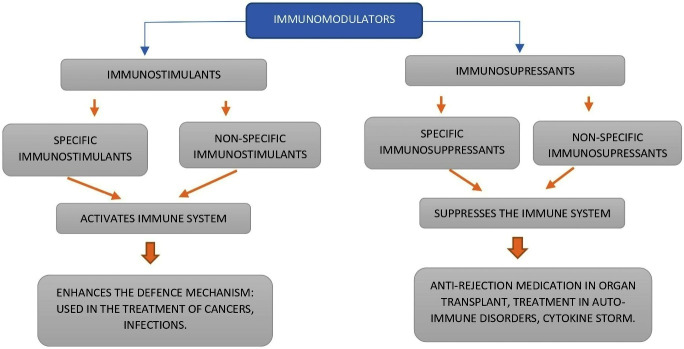
Physiological actions of immunomodulators

**Figure 2:**
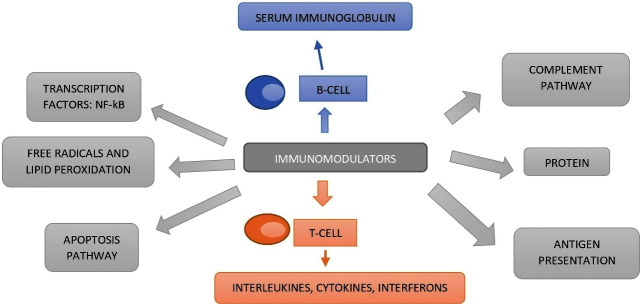
Targets involved for the action of immunomodulators

Immunity is the general ability of a host to resist the activities of foreign bodies or more precisely antigenic compounds. These components of immune system work together to prevent access of pathogens to the functional mechanisms in the body. If any foreign substance or a pathogen is recognized, the coordinated reaction of the host immune system to neutralize or eradicate its effects is defined as an immune response. It involves recognition and defending strategies against the invaders.^1^ Immune system is mainly classified into two types such as innate immunity (non-specific) adaptive or acquired immunity (specific).^2^ The primary defence against human illness is innate immunity through pathogens or external substances. Innate immunity primarily involves infection elements like skin, mucous membrane, stomach acidity. Blood proteins such as inflammatory proteins and cytokines which regulate the immune cells.^3^ Thus, immune system activates the immune response by these components if any invader, pathogen or foreign substance enters into body.^4^ The immune system also gives signals when infection has arisen and enhances adaptive immunity consequently.^5^ Lymphocytes including B-cells and T-cells and their secreted antibodies are essential components of adaptive immunity. B-cells are also known as plasma cells which play a crucial role in humoral immune system. B-cells secrete huge amounts of specific antibodies to fight against foreign antigens or invaders.^6^ Lymphocyte T-cell mainly involves cell mediated immunity and are most effective in the destruction of intracellular bacterial cells, virus infecting cells.^7,8^ The important aspects of immunomodulatory actions are immunostimulation (strengthening of immune system), immunosuppression (suppresses the immune system) and as immunoadjuvants. Hence, both immunostimulants and immunosuppressants have their own role in eliciting the anticipated activity. An overactive immune system cannot distinguish itself (normal immune cells) from the non-self (foreign antigens) in auto-immune disorders and contributes to the loss of self-entity (normal immune cells). Here, immunosuppressants play a vital role in moderating the immune system to near normal functionality. They also play a vital role in reloading the deficiency of immune system as seen in the infections like AIDS (Figure 1). On the other hand, vaccine adjuvants, for example, Freud’s adjuvant, may increases the activity of vaccines. Immunomodulators can also induce different cellular activities such as apoptosis, free radical and lipid peroxidation, protein synthesis etc. They also, target various immune mediators and transcription factors which are shown in (Figure 2). A large body of evidence exists in the realm of scientific literature exploring the Indian traditional plant secondary metabolites for immuomodulation.^9,10^ This review is an attempt to analyse the role and current status of various immunomodulators from plant origin.

## Plant derived immunomodulators

Three quarters of population depend on herbal medicine according to the World Health Organization (WHO).^11^ For the prevention, cure of microbial and life style diseases, plants and minerals have been used since the millennial. Medicinal plant and their extracts trigger the immune system to assist in a therapeutic approach. Plant extracts and various active constituents are noticed to protect the host immune system and majority of plants includes in this class. They play a crucial role in the treatment of infections, inflammation and in immunodeficiencies by their effects on various cells via interleukins, cytokines. The general mode of action is through immunostimulants, immunosuppressants, or by immunoadjuvants to enhance antigen specific immune response.^12^ Traditional Indian system medicines like Siddha, Unani, Ayurveda enhance the body natural resistance to the disease and strengthen the body’s natural disease resistance. A variety of Indian medicinal plants have been used for own immunomodulatory response, such as various ‘rasayana’. In addition to rasayana, plants are classified as non-rasayana which also exhibit anti-inflammatory, immunostimulatory, anti-pathogenic activity.^13,14^ The herbs have immunomodulatory activity in both rasayana and non-rasayana are presented in Table 1.

**Table 1:** Rasayana and non-rasayana plant immunomodulators

**Rasayana plants**	**Non-rasayana plants**
*Acorus calamus*	*Andrographis paniculata*
*Allium sativum*	*Butea monosperma*
*Aloe vera*	*Calotropis procera*
*Asparagus racemosus*	*Gymnema sylvestre*
*Azadirachta indica*	*Mangifera indica*
*Boerhavia diffusa*	*Mentha spicata*
*Curcuma longa*	*Ocimum sanctum*
*Piper longum*	*Saraca indica*
*Tinospora cordifolia*	*Viscum album*
*Withania somnifera*	*Zingiber officinale*

 Numerous studies have been performed on the effects of plant constituents on the immune system, which include rasayana as well as non-rasayana plants. The focus of this review is on several essential medicinal plants and immunomodulators derived from plants, and their constituents towards immunomodulation.

###  Various plants used as immunomodulators

####  Curcuma longa

**Figure 3:**
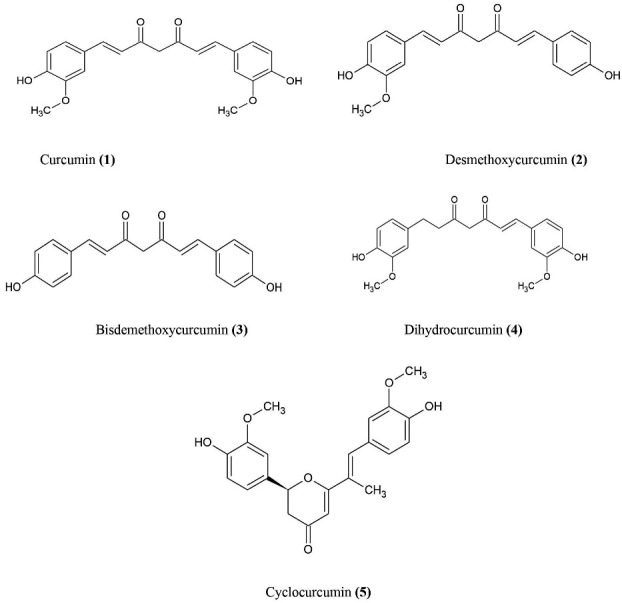
Chemical structures of constituents of curcumin longa

In *Curcuma longa* (Turmeric), curcumin is active innards of the turmeric which is used as nutritional spice for our daily use (Figure 3). Curcumin is widely extracted from rhizomes of *C. longa*, belongs to *Zingiberaceae* family. The plant is mainly distributed to Southeast Asia, has intense bright yellow to golden colour and a peppery, bitter taste. The alternative name of turmeric is Indian saffron. *C. longa* has wide diversity of curative and pharmacotherapeutic application since decades.^12,15^ The plant can also be used for treating diseases such as carcinoma, inflammation, microbial infections, diabetes, arthritic, muscular disorders, biliary disorders, anorexia, cough, diabetic wounds, hepatic disorders and sinusitis.^16^ Studies have shown that turmeric mainly contains proteins, fats, carbohydrates, minerals, volatile and non-volatile oils and curcuminoids. Curcuminoids is an amalgam of curcumin **(1)** (60-70%), desmethoxycurcumin **(2)** (20-27%), and bisdemethoxycurcumin** (3)** (10-15%). and little amounts of dihydrocurcumin **(4)**, cyclocurcumin **(5)**. Other than curcuminoids, sesquiterpenes, diterpenes, triterpenoids, turmeronesare also present in the plant.^17^ Normally, uptake of curcumin leads to immunomodulation in the body through the interaction of cellular components (dendritic cells, macrophages, B and T cells), molecular components (cytokines, transcription factors, downstream signalling pathways), and in the inflammatory responses.^18^ It decreases the inflammation by blocking the development of the NO production, cyclooxygenase-2 (COX-2), nuclear factor kappa B (NF-κB), inducible nitrogen oxide synthase and lipoxygenase in NK (natural killer) cells and IFN (interferon)-gamma, or tumour necrosis factor-alpha (TNF-alpha) enabled macrophages, causes curcumin to reduce inflammation. Some studies report that phosphorylation of 1 kappa B alpha blocks Nf-kB activation in cell lines such as phorbol 12-myristate 13-acetate (PMA) and 12 H_2_O_2_ human myelomonoblastic cells.^16,19^ The tumour development in the body is mediated through AP 1 and NF-κB transcription factor. Proliferation and survival of the cell is activated by PMA which is regulated by protein kinase C. Protein kinase C is also activated by lipopolysaccharide (LPS) and TNF-alpha which activates NF-κB.^20^ Consequently, curcumin inhibits protein kinase C which weakens the Nf-kb activation. [Anti-inflammatory activity shown by blocking the activation of Nf-kb transcription factor and activator protein (AP 1)]. Activation of TNF-alpha suppresses the activator protein 1 in bovine aortic endothelial cells. Uptake of 25 µM curcumin reduces NF-κB and AP 1 binding. Proinflammatory cytokines such as TNF-alpha, IL-1, IL-6, IL-12 secreted by active immune cells were blocked by induction of curcumin which are responsible in inflammation through LPS or PMA-stimulated monocytes, macrophages, dendritic cells, and splenic lymphocytes.^21^ After the treatment of curcumin in high concentration reduces the binding activity of STAT3 with DNA, phosphorylation of STAT3, and the expression of IL-1beta, TNF-alpha for some histological injury. On the other hand, treatment of curcumin in low concentration shows anti-inflammatory activity and show an enhancement of STAT3 phosphorylation. Therefore, based upon the concentration of curcumin shows dual activity on STAT3 modulation.^22,23^ Absence of curcuminoids in the polysaccharides of polar extract in curcumin, these polysaccharides triggers mitosis and seen elevated levels of splenocytes contrast to LPS. In unrestored murine macrophages and splenocytes seen elevated levels of IL-2, IL-6, IL-10, IL-12, IFN-gamma, NO, MCP-1, TNF-alpha by induction of purified polysaccharides.^24^ We conclude curcumin and other chemical constituents of *C. longa* have capacity to stimulate the immune response. Other than curcuminoids turmerin, elemene, furanodiene, curdione, bisacurone show anti-inflammatory and anti- cancer activities. They are not toxic, well tolerated, flavour sweet taste.^21^

####  Andrographis paniculata

**Figure 4:**
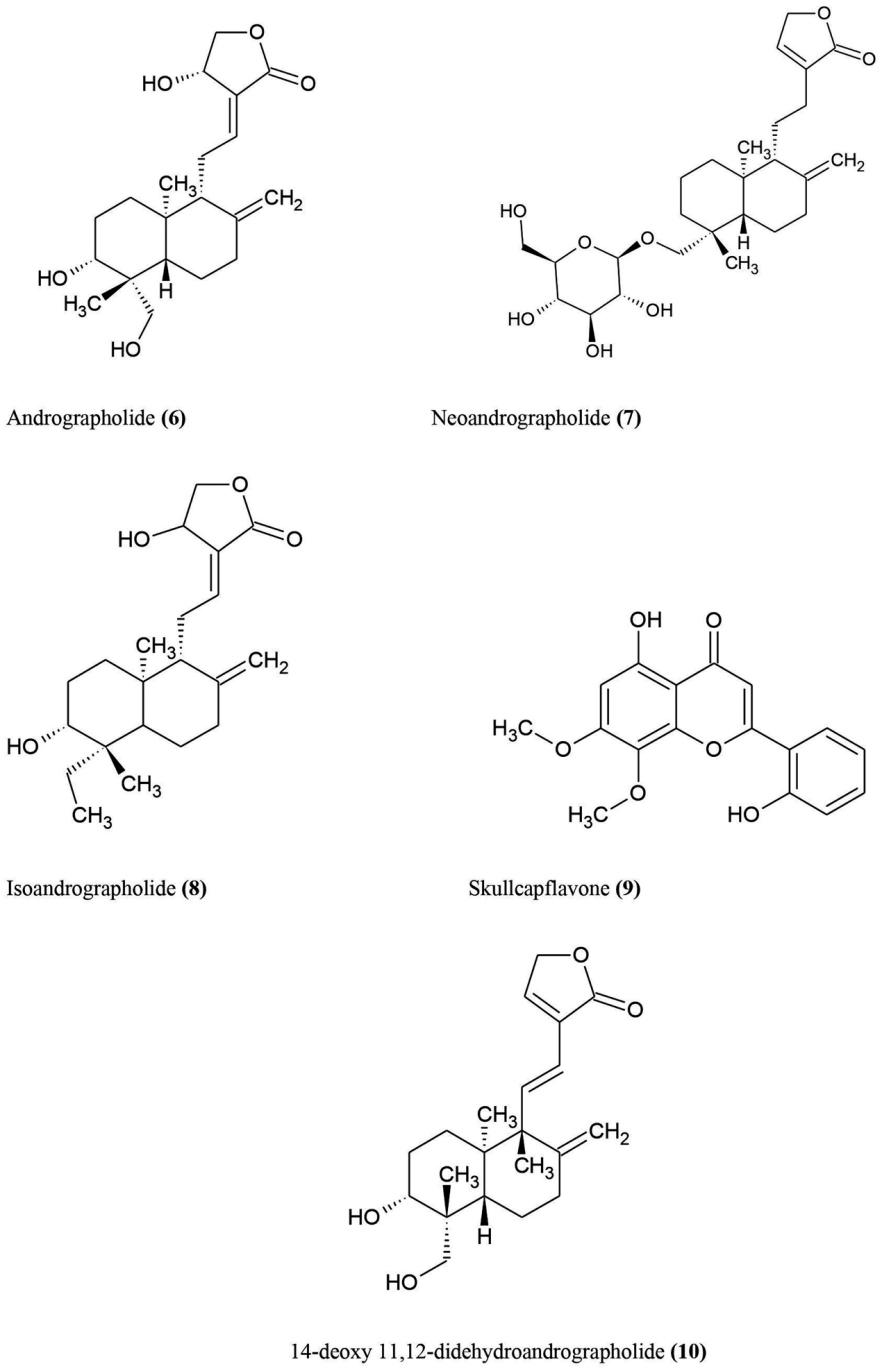
Chemical structures of constituents of *Andrographolide paniculata*

*Andrographis paniculata* traditionally called as King of Bitters, Kalmegh, is a medicinal plant belongs to *Acanthaceae* family. It is widely distributed through Southeast Asia, China, West Indies, America, Thailand, Malaysia, and Japan. It has bitter taste and colourless appearance. *A. paniculata* is used in the world as ayurvedic and homeopathic medicine, consisting mainly flavonoids (flavone), polyphenols, terpenoids (diterpene, lactones, ent-labdane), xanthones, nocordioides.^12^ Several andrographolides and their derivatives have been demonstrated for immunostimulatory behaviours in two ways in recent decades: (1) Antigen specific response (antibodies are insane to keep bacteria entering), (2) Non-specific immune response (macrophage cell scavenger and capture invaders). Andrographolides and their derivatives also exhibit antiviral, anti-bacterial, anti-inflammatory, anti-platelet aggregation, anti-diabetic, anti-tumour, anti-microbial, hepatoprotective. Andrographolide **(6)** is the major chemical constituent in *A. paniculata* which is found mainly in leaves.^25^ It is chemically known as 3-alpha, 14, 15, 18-tetrahydroxy-5-beta, 9-betaH, 10-alpha-labda-8, 12-dien-16-oic acid gamma-lactone. *In vitro* studies of andrographolide show that, andrographolide decreases IL-12, TNF-alpha, NO, PGE2, COX-2, inducible nitric oxide synthase (iNOS) in microglia and macrophages, and also block LPS-induced iNOS and COX-2 bearing in RAW264.7 murine macrophage cell lines.^21^ Increasing the cytotoxic ability of lymphocytes is also seen in improving CD markers and the construction of TNF-alpha by andrographolide administration.^26^ Some other studies say that andrographolide enhances the antibody production and multiplication of human peripheral blood lymphocytes, cytokines and decreases delayed type hypersensitivity response.^27^ From the studies of insulinoma tumour, andrographolide shows crackdown of proinflammatory proteins bearing iNOS and COX-2 by blocking NF-κB binding to DNA, STAT-3 by down-regulation of cytokine 1 and 3 signalling. The andrographolide shows better activity on signalling pathways like TLR4/NF-κB for insulinoma tumour^28,29^ and possess anti-cancer studies such as enhancement of cell differentiation, IL-2, IFN-c, tumour suppressor proteins P53 and P21. It also down regulates the cancer cell replication, relocation, cell-cycle arrest at G2/M phase and decreased E-selectin expression, JAK/STAT/NF-κB signalling pathway.^5,27,30,31^Anti-viral studies of andrographolide shows enhancement of CD4+ T cells against HIV-1 infection and blocks dysregulation of cell cycle.^32^ They block the HIV virus by inhibiting HL2/3 adhesion to TZM-bl cells through andrographolide association between gp20 and CD4, CCR5, CXCR4.^33^ Other studies show down-regulation of herpes simplex virus, Epstein-Barr virus, Dengue virus 1, flavivirus and Pestivirus by andrographolide. Along with andrographolide, other constituents (Figure 4) such as neoandrographolide **(7)**, isoandrographolide **(8)**, skullcapflavone **(9)** which are mainly used to reduce inflammation by blocking inflammatory cytokines like IL-6, NO, IL-1beta in stimulated LPS- macrophages.^12^ Andrographolide and 14-deoxy 11, 12-didehydroandrographolide **(10)** show anti-oxidant activity by free radical scavenging effect and lipid peroxidation blocking impact.^34^ Some experimental studies show that there is a significant enhancement of catalase, superoxide dismutase, glutathione-s-transferase subsequently decrease lactate dehydrogenase, thiobarbituric acid reactive substance.^27,35,36^ Some evidences show that the extract of andrographolide reduces the intensity and duration of cold.^37^ From a study, we came to understand that PHA-induced human peripheral lymphocytes andrographolide increase in lymphocytes multiplication and IL-2 production.^38^ Andrographolide demonstrates reduction in IL-2 development with concanavalin-A *in vitro* in the murine T-cells. Andrographolide also block the production of TNF-alpha, IL-12.^39,40^

####  Echinacea purpurea

**Figure 5:**
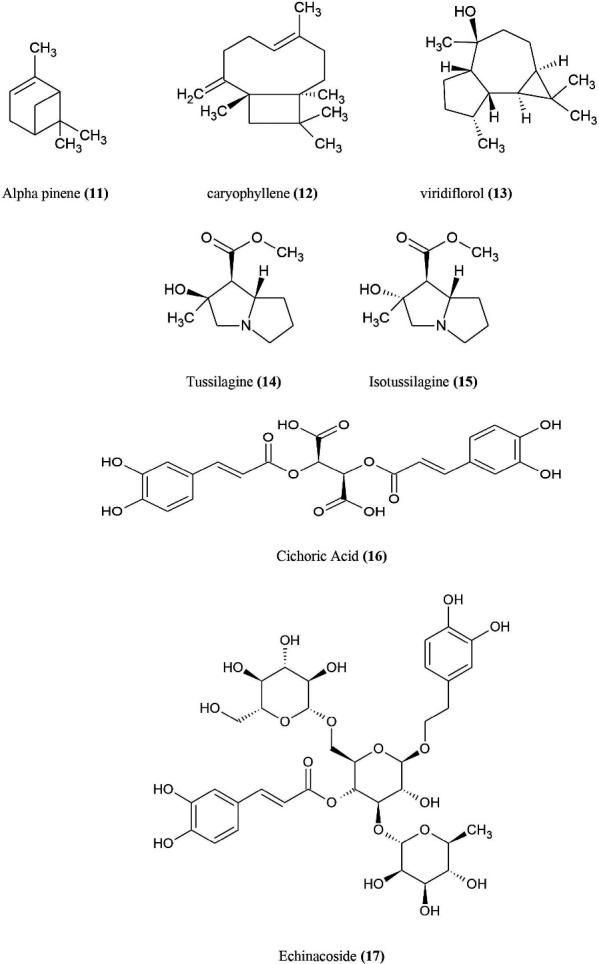
Chemical structures of constituents of Echinacea

*Echinacea purpurea* is traditionally known as coneflowers, *E. purpurea* is perennial medicinal plant containing nine herbaceous flowering plants belongs to the family *Asteraceae*. It is widely distributed in eastern and central north America. *Echinacea* has been used for decades due to its safety and longevity. It shows many benefits.^17,25,41^ *Echinacea* particularly contain three species namely: *E. purpurea, E. angustifolia, E. pallida* has many similar pharmacological effects such as immunostimulatory, anti-inflammatory, upper respiratory tract infections, common cold, bronchitis, cough, sore throats, inflammation in mouth and pharynx, snake and insect bites.^17,25^ *Echinacea* is a dietary medicinal supplement and best seller in Europe and USA, which is low cost and effective natural immunomodulatory effects, with increasing innate and non-specific innate immunity.^42^ It shortens the length of common cold, and possess anti-viral, anti-microbial activities from a series of studies on these species. The active constituents of *Echinacea* (Figure 5) are volatile oils [alpha pinene **(11)**, caryophyllene **(12)**, viridiflorol **(13)**], pyrrolizidine alkaloids [tussilagine **(14)**, isotussilagine **(15)**], caffeic and ferulic acid derivatives [cichoric acid **(16)**, echinacoside **(17)**] and polysaccharides (acidic arabinogalactan, rhamnoarabinogalactans, 4-O-methylglucronylarabinoxylans).^43^ The innate immunity is enhanced by echinacea. Several *in vitro* studies say that *Echinacea* plays a positive role in fighting infections, free radical generation which show anti-oxidant activity. It also enhances the circulating leukocytes, monocytes, replication of phagocytosis in the spleen and down regulation of neutrophils due to migration of granulocytes in the tissue, which in turn activate the immune cells.^17^ It is understood that oral administration of *Echinacea* to normal rats stimulate inflammatory cytokines TNF-alpha, IL-1beta, IFN-Beta, IL-10 and increases T-cell function.^17,44^ In addition, the dried powder of *Echinacea* show enhancement of CD4+ T-cells in blood in mouse when injected intra peritoneally. Alkamides block IL-2 production in human T-cells/Jurkat cells and have an effect on NfkB.^17,45^ *E. angustifolia* block leucocyte 5-lipoxygenase activity and microsomal cyclooxygenase.^17^ Arabinogalactan which is a chemical constituent of *Echinacea* show enhancement in macrophage activation.^44^ *E. purpurea* extracts increase NK-cells in human peripheral blood.^46^ Some other experimental studies show reduction in paw edema formation and inflammation of croton oil ear test by *Echinacea.*^41^ The mice treated with purified polysaccharides of *Echinacea* shows enhancement of immune functions as like mice treated with cyclosporin or cyclophosphamide. This study reveals that use of *Echinacea* produce immune boosting in immunosuppressed animals.^47^ Some of marketed *Echinacea* products namely, Immunal drops (succus of *E. purpurea*), Immunace Forte tablets (*E. purpurea* herbae succus siccum), Echinacea Forte drops (juice squeezed from fresh flowers of *E. purpurea*). Hence *Echinacea* is suggested as a prophylactic medication in winter with no side effects.^41,48,49^

####  Tinospora cordifolia

**Figure 6:**
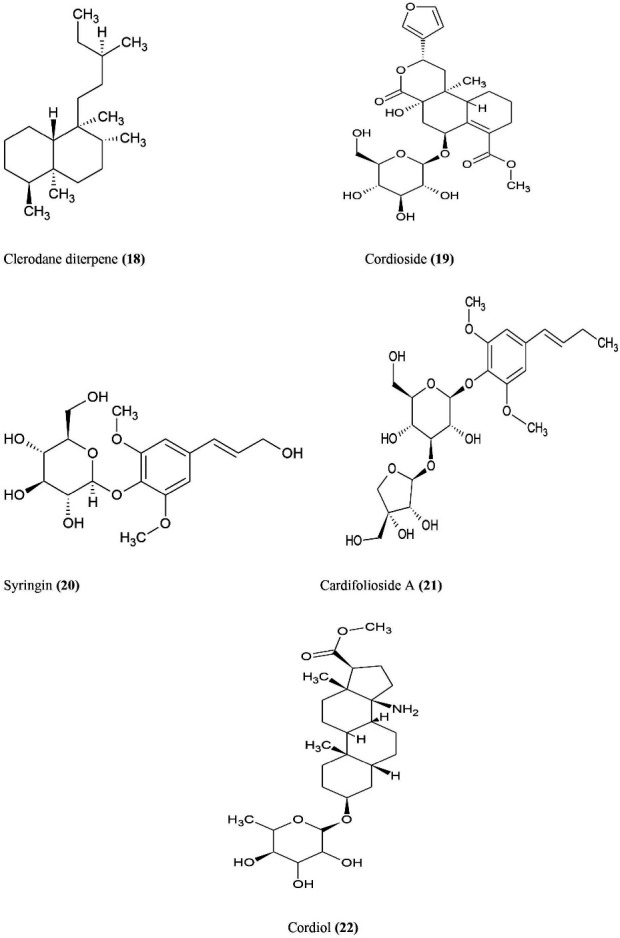
Chemical structures of constituents of *Tinospora cordifolia*

*Tinospora cordifolia* traditionally known as Guduchi in Sanskrit, commonly called as moonseed, giloy which is a biologically, abundant, wide, deciduous, climbing shrub with greenish, typical yellow flowers belongs to the family *Menispermaceae*. *T. cordifolia* mainly found in tropical Indian-subcontinent, China.^12,50^ These plants mainly located at higher altitudes of 300 m.^25^ Extract of *T. cordifolia* show wide spectrum of immunotherapeutic properties ranging from basic tonic, anti-inflammatory, anti-arthritic, anti-malarial, aphrodisiac, anti-allergic, anti-diabetic, anti-hepatotoxic, wound healing effect and anti-pyretic properties are shown and has relatively low toxicity.^50^ *T. cordifolia* is an herbal medicine, annually its preparation utilizes 10 000 tonnes of crude herbal extract.^51^ The active phytochemical constituents **(**Figure **6)** of *T. cordifolia* includes clerodane Furano diterpene glycoside **(18)**, cordioside **(19)**, syringin **(20)**, Cordifolioside A **(21)**, Cordifolioside B, cordiol **(22).**^12,25,50^ Aqueous and alcoholic extract of *T. cordifolia* have been tested and reported successfully as immunomodulatory activity.^52^ In addition, alpha D glucan, extracted from the stem of *T. cordifolia* show immunostimulatory activity.^12^ Higher concentration or dose dependent of *T. cordifolia* show anti-oxidant, anti-complementary, immunopotentiation effects due to augmentation of IgG antibodies. Antibody production is also enhanced due to higher concentration of *T. cordifolia.*^53^ The phagocytic cells measure reactive oxygen species which are generated during anti-inflammatory response.^54^ The ethyl acetate extract of *T. cordifolia*produces the effects like proliferation of stem cells, enhancement of WBCs alpha esterase positive cells, enhancement of antibody producing cells.^55^ The leaf extract of *T. cordifolia* shows action against many bacterial infections such as* E. coli, Staphylococcus aureus, Streptococcus pyogenes, Bacillus subtilis* and* Proteus vulgaris.*^51^ *T. cordifolia* is indeed useful of tumour growth reduction when compared with cyclophosphamide. It also enhances the humoral and cell-mediated immunity by activation of B cells and T cells when innate immunity fails. Overall, it boosts total immune system by defence mechanism and well-being of host.^12,55^

####  Azadirachta indica

**Figure 7:**
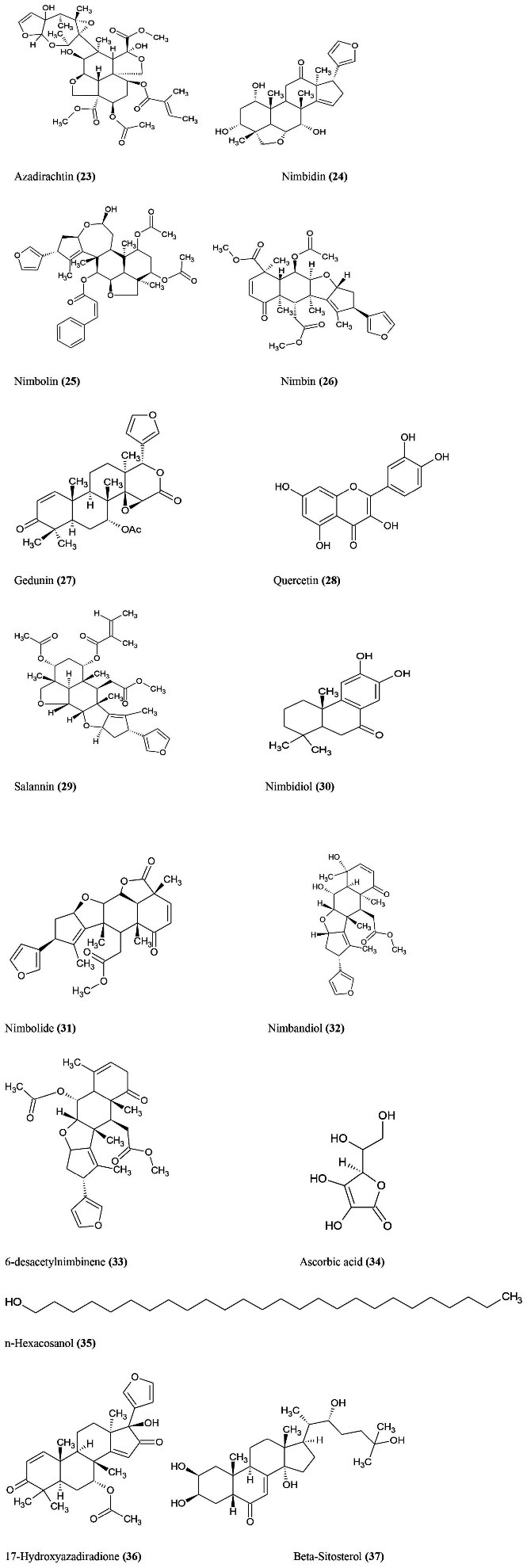
Chemical structures of constituents of *Azadirachta indica*

*Azadirachta indica* traditionally known as neem which belongs to the family *Meliaceae*, which is widely distributed throughout India, Pakistan, Bangladesh, Nepal.^12,25,56^ Neem is a powerful health promoter as well as powerful immune booster because of its rich source of anti-oxidants.^25^ It is used as a medicament and prevention of various diseases in worldwide especially Indian sub-continent as Ayurveda and Unani medicine.^56^ Neem leaves are compound, imparipinnate consists of 5-15 leaflets. Neem tree is about 20-23 m long, with a straight trunk is present. The fruits are golden yellow colour on ripening.^57^ *A. indica* has many properties of pharmacology such as anti-oxidant, anti-inflammatory, anti-cancer, immune booster, anti-diabetic, anti-viral, anti-bacterial, anti-fungal, hepatoprotective, anti-malarial, anti-nephrotoxicity, wound healing effect.^56^ *A. indica* shows pharmacological role in health care due to large sources of active constituents (Figure 7). The main active constituents are azadiracthin **(23)**, nimbidin **(24)**, nimbolin **(25)**, nimbin **(26)**, gedunin **(27)**, quercetin **(28)**, salannin **(29)** and nimbidol **(30)**. Neem leaves also contain some active constituents such as nimbolide **(31)**, nimbandiol **(32)**, 6-desacetylnimbinene **(33)**, ascorbic acid **(34)**, n-hexacosanol **(35)**, 17-hydroxyazadiradione **(36)**, beta-sitosterol **(37)**, polyphenolic flavonoids.^56,58,59^ Primarily neem shows anti-fungal and anti-bacterial activity due to the presence of quercetin, beta-sitosterol and polyphenolic flavonoids.^56^ Neem stimulate the immune system through macrophage activation (CD25 & MAC-3), enhancement of white blood cells, CD4+, CD8+, T-cells.^60^ Neem oils activates cell-mediated immunity to obtain an increased response of antigenic challenges.^61^ NIM-76 a compound of leaf extract shows activation of polymorphonuclear leukocytes count, stimulate macrophage, increase in T-cell responses.^62^ When crude neem oil is injected to mice, stimulate T-cells to obtain gamma interferon and activates macrophage.^63^ Azadirachtin and nimbolide shows free radical scavenging activity.^25^ The chemical constituents of neem modulate various signalling pathways like tumour suppressor genes (enhancement of p53, pTEN genes), angiogenesis (decrease of vascular endothelial growth factor), transcription factor (reduction of NF-kB), and apoptosis (increase BCL2, decrease of Bax) to inhibit cancer formation and progression.^56^ *A. indica* (2 g/kg) is used for the treatment of new castle disease virus antigen by enhancing antibody production.^64^ The active constituents of neem reduce parasitaemia and show inhibitory effect on microbial growth of cell wall cleavage. Neem also plays a very important role in anti-inflammatory responses via COX (cyclooxygenase) and LOX (lipoxygenase) pathways.^56^ Leaf extract of neem administered orally enhances IgM and IgG levels and also increases anti-ovalbumin antibody.^25^

####  Allium sativum

**Figure 8:**
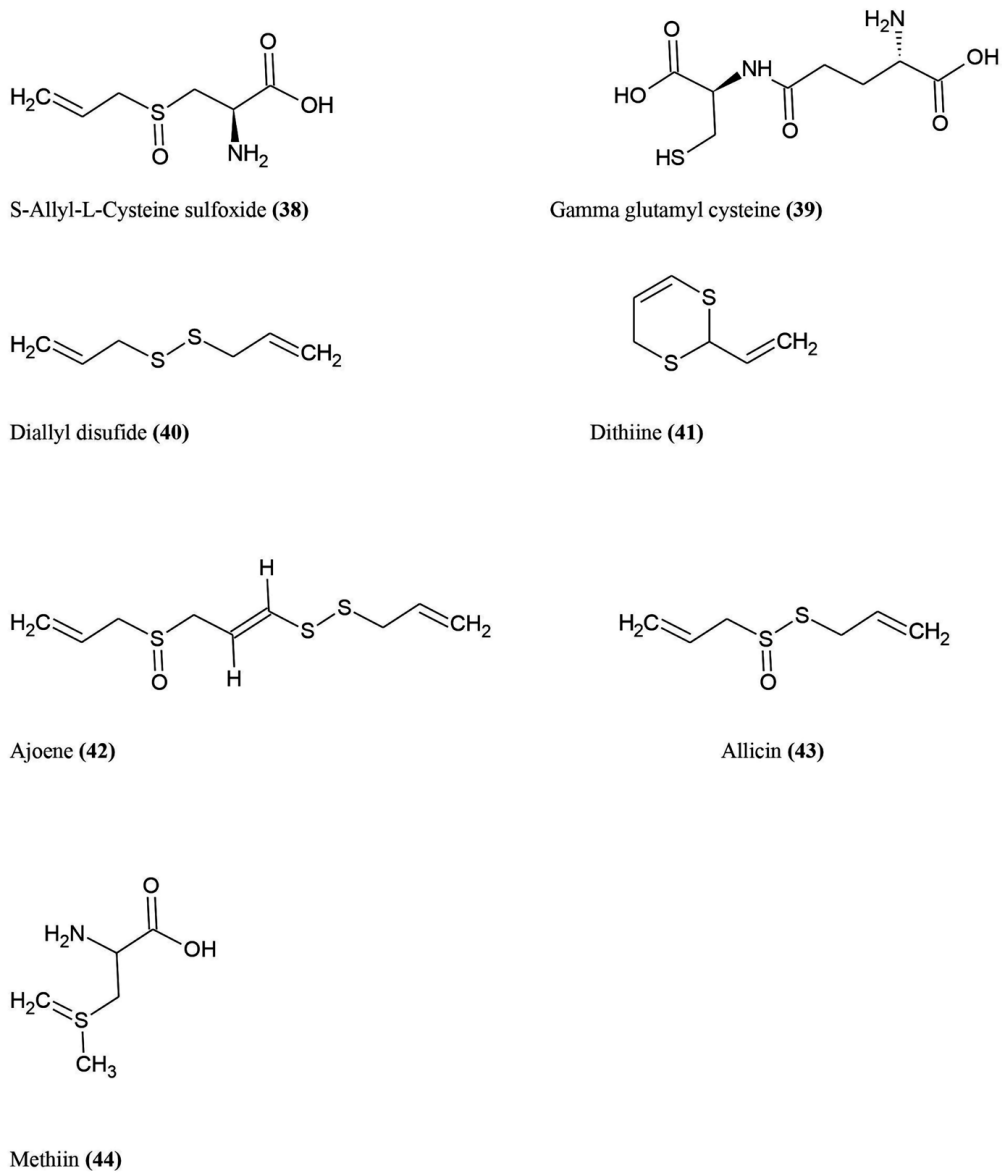
Chemical structures of constituents of *Allium sativum*

*Allium sativum* commonly known as garlic which is an onion species belongs to the family *Alliaceae*. *A. sativum* is widely distributed in central Asia (native) to worldwide. *A. sativum* is one of the most used plant as essential medicinal spice and dietary medicament and also used as traditional medicine attributed with immunomodulatory properties for over 6000 years ago.^65^ At present garlic is used as individual medicine in all over the world to treat and prevent certain diseases. As we see our history, garlic is used for many prophylactic and pharmacological benefits. The biological activity of garlic shows free radical scavenger, cardiovascular diseases, immune stimulant, anti-cancer, anti-inflammatory, anti-infectious diseases, anti-oxidant, anti-allergic, anti-bacterial properties.^65,66^ Garlic extract contains more than 200 diverse chemicals and also possesses high concentration of sulphur which is mainly responsible for health benefits and flavour. Garlic mainly contains water, carbohydrates, proteins, organosulphur compounds, fibres.^67^ The bioactive chemical constituents (Figure 8) of garlic mainly include S-allyl-L-cysteine sulfoxide (alliin) **(38)**, gamma glutamyl cysteine **(39)**, Diallyl Disulphide (DADS) **(40)**, Dithiines **(41)**, (E-Z) ajoene **(42)**, diallyl thiosulphinate (allicin) **(43)**, methiin **(44)**. The few studies show that in vitro influenza A and B are shown by garlic extract.^68^ Different compounds and formulations show immunomodulatory effects including cytokine secretion, regulation, stimulation of phagocytosis, macrophage activation, immunoglobulin production.^66^ Garlic preparations are mainly liquid preparations such as (aqueous, oil or solvent extracts), solid (dried garlic powder, fresh cataplasm).^69^ Alliin is bioactive chemical constituent which is extracted from allium sativum show enhancement of IL-6, MCP-1 (pro-inflammatory cytokines). At low concentration, garlic extract show cytokine modulation which include increase of IL-10, and decrease of IL-12, IL-1alpha, IFN-gamma, TNF-alpha, IL-6, IL-8.^70^ Other active constituents of garlic allitridin, S-allyl-L-cysteine, caffeic acid, uracil, diallyl sulphide inhibits transcription factors NF-KB, IL-6, MCP-1, TNF-alpha, IL-1beta, IL-12.^71^ Allicin is administered to mice infected by *Plasmodium yoelii* show reduced parasitaemia due to stimulation of proinflammatory cytokines like IFN-gamma, macrophage activation, CD4+ T-cells, CD40, maturation of dendritic cells.^72^ Oil macerated extract contains Z-ajoene which stimulate B-cells and T-cells, and enhances interleukins and IgA antibodies. Aged garlic show anti-tumour, decreases IgE mediated skin reactions, and also show anti-cancer activity by affecting NK-cells. Garlic extract enhances IL-10 on monocytes and decrease TNF-alpha on LPS-stimulation leads to anti-inflammatory activity, and also stimulate CD8+ T-cells which leads to delayed type hypersensitivity response.^12^

####  Boerhaviadiffusa

**Figure 9:**
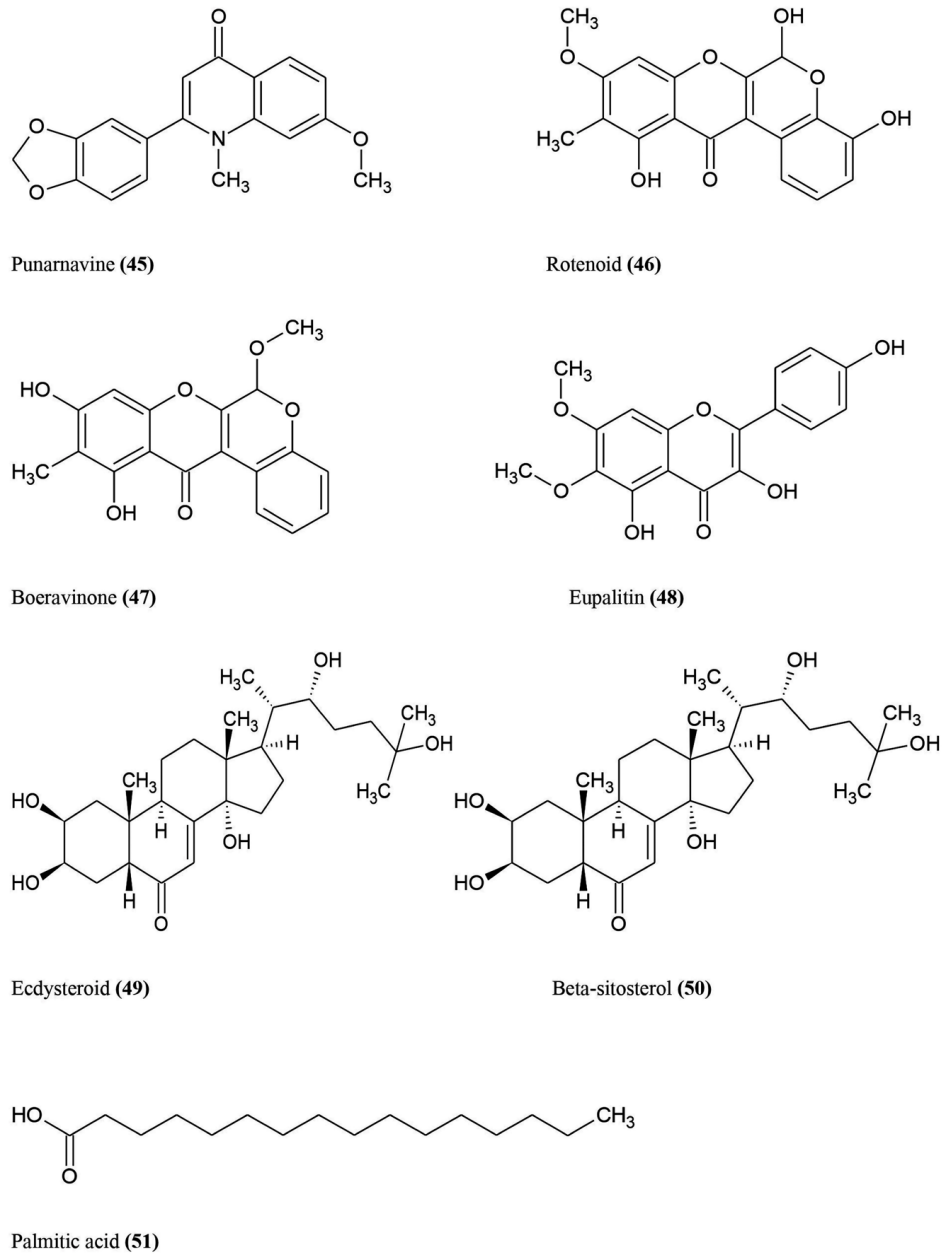
Chemical structures of constituents of *Boerhavia diffusa*

*Boerhavia diffusa*, a perennial herb commonly called as Punarnava, raktapunarnava, shothaghni, kathillaka, kshudra, tarvine, varshabhu, raktapushpa, varshaketu, red spiderling and shilatika in India, which belongs to the family *Nyctaginaceae. B. diffusa* is widely found in Asia, Africa, North America, South America and Caribbean.^12,25,73,74^ Punarnava is a major therapeutic herb in Ayurveda which restores youth and has rejuvenating property. Punarnava is arisen from two terms, one is punar (once again/regaining/restoring) and another is Nava (new, renew, young).^74^ Punarnava contains some bioactive chemical constituents **(**Figure **9)** mainly punarnavine **(45)**, isoflavonoids (rotenoids) **(46)**, sitosterol, alkaloid (boeravinone) **(47)**, Eupalitin **(48)**, ecdysteroid **(49)**, beta-sitosterol **(50)** and palmitic acid **(51).**^75^ These active chemical constituents are found in various parts of plant that show pharmacological and therapeutic properties which includes immunomodulatory, anti-inflammatory, anti-diabetic, anti-oxidant, anti-viral, anti-fungal, anti-microbial, anti-stress, diuretic, anti-fibrinolytics, anti-convulsant, hepatoprotective, renoprotective, anti-histamine, adaptogenic, laxative, anti-metastatic.^74^ The plant extract of punarnava mainly helps in the treatment of discharge of fluids (dropsy) in tissues and body cavities.^75^ The plant extract contains alkaloid fraction which enhances or normalize the plasma cortisol and reduce the delayed type hypersensitivity reaction and increase antibody titre in animals when administered intraperitonially 25-100 mg/kg for 10 days.^76^ Pure alkaloid (40 mg/kg) punarnavine stimulate immune system, which enhances the WBC count, splenocytes, thrombocytes, bone marrow cells, alpha esterase positive cells. Administration of punarnavine simultaneously leads to suppress lung melanoma metastasis upregulates of IL-2, IFN-alpha, NK-cells, ADCC (Antibody-dependent cellular cytotoxicity) and down regulates of proinflammatory cytokines like IL-1beta, IL-6 and TNF-alpha.^77^ Alkaloid fraction remarkably reimpose the suppressed humoral response in stressed rats.^25^ Some studies show that *B. diffusa* compared with drug levamisole, up regulates the phagocytic activity and macrophage activation.^78^ Ethanolic extract of *B. diffusa* shows immunosuppressive activity in murine macrophages by inhibiting the NK cell cytotoxicity in human and suppress the production of LPS induced nitric oxide at low concentration (10 µg/mL). Stimulated TNF-alpha in human peripheral blood mononuclear cells and stimulates IL-2.^12^ The active chemical constituent of ethanolic extract is Eupalitin-3-O-beta-D-galactopyranoside. Immunosuppressive property of Eupalitin is attributed to anti-osteoporotic properties and also used for the treatment of rheumatic disorders.^73,74^ Methanolic extract of Punarnava inhibited cell viability. In MCF-7 cell lines studies, cells have been arrested in Go-G1 phase and also inhibit the metastasis in mice B16F10 melanoma. Punarnava is mainly used as a prophylactic for various disorders in humans and animals.^12^

####  Acacia catechu

**Figure 10:**
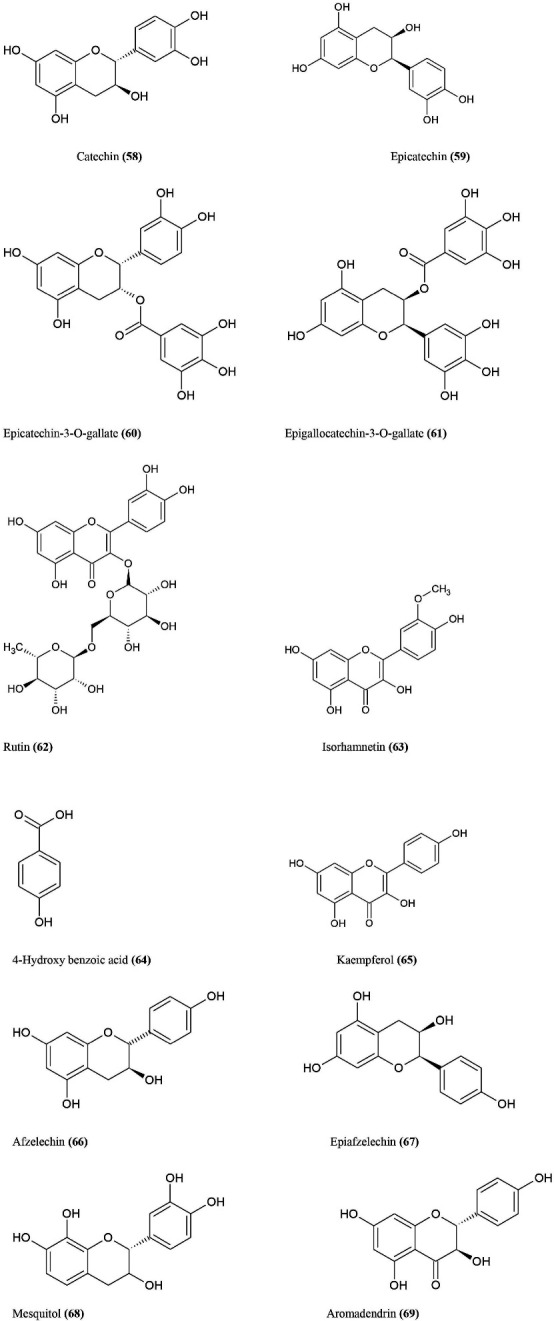
Chemical structures of constituents of *Acacia catechu*

*Acacia catechu* is commonly called as cutch tree, black catechu, cachou belongs to the family *Fabaceae*. It is widely distributed in India, other Asian countries and east Africa.^12^ *A. catechu* has several names in different languages which are kattha (Urdu), khadir (Hindustani and Punjabi), khoyer (Bengali), khair (Hindi), kaath (Marathi), kachu (Malay). *A. catechu* contains nearly 1300 species which are used as a medicament, demulcent, emulsifier, food spice, all over the India from past 2500 years. *A. catechu* has many pharmacological properties in different parts of *A. catechu* which are heartwood, bark, leaves, seeds, seedpods. Therapeutically *A. catechu* is used as anti-microbial, immunomodulator, anti-inflammatory, anti-fungal, coagulant, vermifuge, anti-diarrheal, astringent, lepromatous leprosy.^79^ The active chemical constituents **(**Figure **10)** are extracted from *A. catechu* such as catechin (polyhydroxylated benzoic acid) **(58)**, epicatechin **(59)**, epicatechin-3-O-gallate **(60)**, epigallocatechin-3-O-gallate **(61)**, rutin **(62)**, isorhamnetin **(63)**, 4-hydroxy benzoic acid **(64)**, kaempferol **(65)**, afzelechin **(66)**, epiafzelechin **(67)**, mesquitol **(68)**, aromadendrin **(69).**^80,81^

Pro inflammatory eicosanoids are suppressed by flavonoids which are extracted by *A. catechu*.^12^ The *in vitro* screening shows anti-oxidant property and protect against DNA strands break and also effective against superoxide anions, hydroxy radicals, anti-lipid peroxidative activity and also possess anti-microbial properties.^82^ In addition, methanol and hexane extract studies have provided the best results for developing better anti-cancer medicines because they have no effect on human peripheral lymphocytes and antiproliferative function.^12^ *In vitro* findings indicate cytotoxic activity for breast adenocarcinoma in MCF-7 cell lines using 70 percent methanol extract from acacia catechu. Flow cytometric studies and morphological investigations also show induced apoptosis. The analysis of immunoblots showed that apoptosis had been induced by the increase in Bax/BCL2 levels which resulted in caspase activation and the subsequent breakdown of polyadeno ribose polymerase.^83^ Administration of aqueous extract of *A. catechu* show immunomodulatory activity on cell mediated immunity and humoral immunity in mice. It also shows significant improvement of phagocytic index, of neutrophil adhesion to nylon fibers, significant protection to neutropenia, upregulation of serum immunoglobulin levels and haemagglutination titre values and downregulation of mice mortality rate.^84^ Based on the concentration, the aqueous extract of acacia catechu show anti-diabetic activity and antinociceptive activity.^12^ Epigallocatechin-3-gallate decreases apoptosis, inhibit the COX-2 expression, proteasomal degradation, pathway of MAPK, DNA methyl transferase 1, topoisomerase II and telomerase which impact the role of chromatin.^21^

####  Panax ginseng

**Figure 11:**
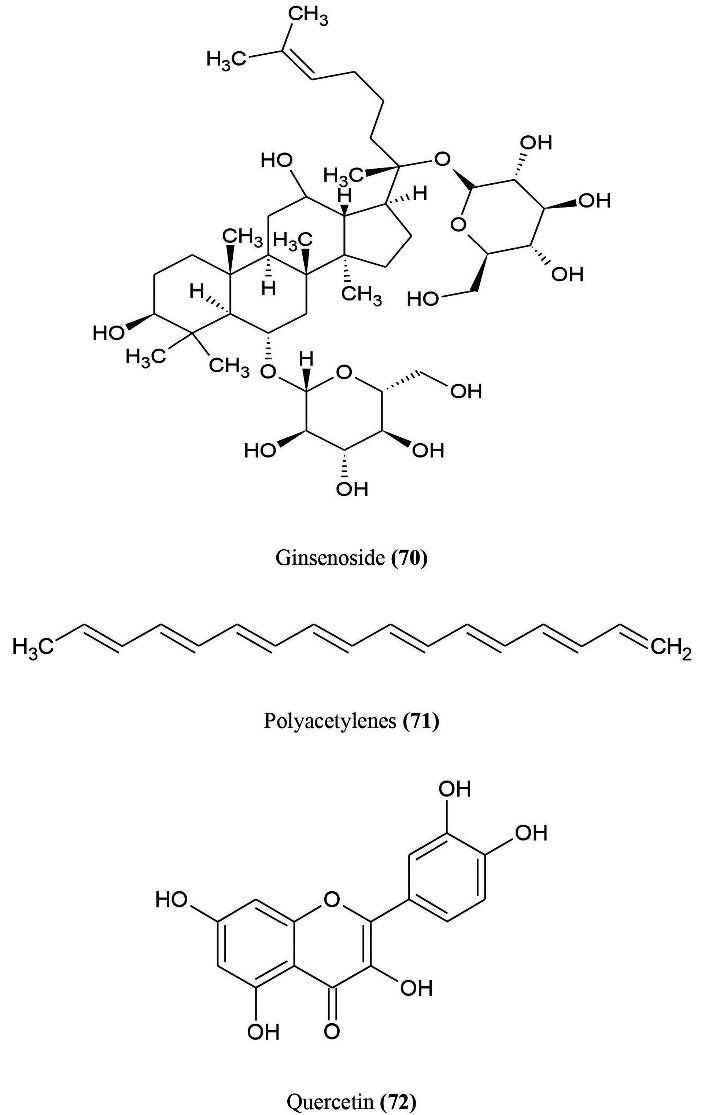
Chemical structures of constituents of Panax ginseng

*Panax ginseng*, traditionally known as ginseng belongs to the family *Araliaceae*. Ginseng is widely distributed in India, Asian countries. One of the best-known medicinal plant and immunomodulator herb is *P. ginseng.* It is generally used as a treatment for different disorders.^12,85^ The active chemical constituents (Figure 11) derived from the ginseng consist mainly of a variety of saponins, tetracyclic triterpenoids saponins (ginsenosides) **(70)**, polyacetylenes **(71)**, poly phenolic compounds (quercetin) **(72)**, acidic polysaccharides.The whole plant (roots, stems, leaves) and their extracts are useful to boost immune system and balance immune homeostasis and improve microbial resistance by immune system responses.^86^ Ginseng is a diverse compound; upon treatment of ginseng every immune cell will respond.^85^ Oral administration of ginseng polysaccharide extracts improves phagocytic activity of peritoneal macrophages.^86^ Ginseng stimulate the production of nitric oxide. Along with ginseng polysaccharides and IFN-gamma also administered leads to enhancement of TNF-alpha, IL-1beta, nitric oxide from macrophages.^87^ J774.1A cell line is used to activate NK-cells and T-cells by IL-12 upon treatment murine macrophage cells with ginseng extract.^88^ *Ginseng* exhibit immunostimulatory activity in dendritic cells reported for enhancement of maturation markers of DC (MHC class II, CD80, CD83, CD86) and also increased IL-1, TNF-alpha production.^89,90^ It also reduces maturation markers of DC (CD40, CD86, CD1a, HLA-DR) and reduces TNF-alpha, IL-12 and IL-12 p40 (a subunit component of IL-12) secretions.^91,92^ Ginseng also show adaptive or acquired immunity, when administered orally or intraperitonially shows formation of IgA, IgM, IgG and IgG1 subunits.^85,93^ Ginsenosides responded H3N2 influenza virus by enhancement of specific IgG, IgG1, IgG2a, IgG2b, and also reported enhanced serum antibodies specific against *Toxoplasma gondii.*^94^ when injected in mice subcutaneously. Ginseng also effects on cell-mediated immunity by enhancing antibody dependent cell cytotoxicity, stimulation of T-cell multiplication, promotes the generation of immunosuppressive regulatory T cells.^12^ Upon treatment, *ginseng* radix produces proinflammatory cytokines like TNF-alpha, IL-6, IL-1beta and INF-gamma by macrophages.^93^ Ginseng and ginsenoside injected to mice reduce TNF-alpha remarkably and show anti-arthritic activity. They also prevent staphylococcus induced sepsis by reducing proinflammatory cytokines via TLR signalling pathway.^95^ Ginseng also show anti-inflammatory, anti-bacterial, anti-microbial, anti-viral, anti-arthritic activity. It is also used as an adjuvant and plays a major role in vaccine formulations by enhancing immunogenicity with supplied antigens.^85,96^ Adjuvant mixed drugs enhances antibody production and activate Th1 and Th2 immune responses.^85,97^

####  Moringa oleifera

**Figure 12:**
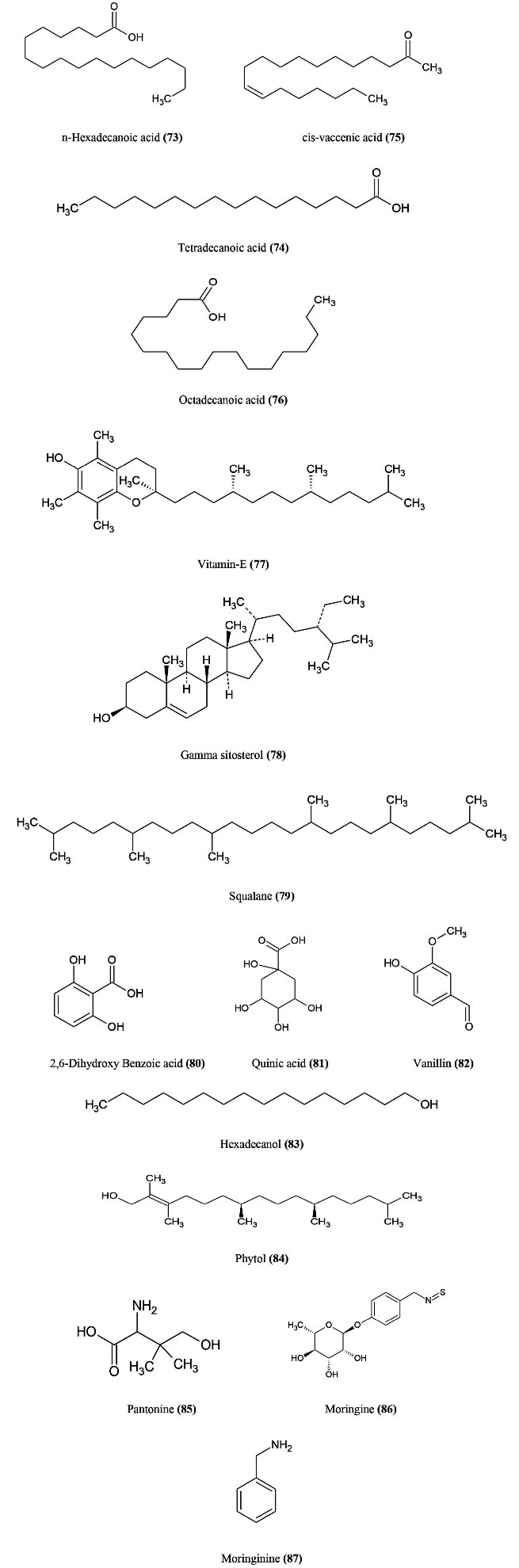
Chemical structures of constituents of *Moringa oleifera*

*Moringa oleifera*, traditionally referred to as a drumstick tree or horseradish tree belongs to the family *Moringaceae*.^98,99^ *M. oleifera* is indegenious to India, Pakistan, Asia, Africa, Afganisthan, Nepal, Bangladesh, Sri Lanka, West Indies, Philippines.^99^ *M. oleifera* is not only utilized for its nutritional qualities, it is also used for medicines. *M. oleifera* is the fast growing plant and rich in nutritious and rich in ammino acids, beta-carotene, vitamin E, vitamin C, polyphenols, antioxidant.^98^ This is known for the African folk medicine and was described in Charaka Samhitha 5000 years ago.^99^ Therapeutically *M. oleifera* is used to treat anti-cancer, anti-inflammatory, hepatoprotective, neuroprotective, anti-diabetic, anti-rheumatoid, anti-fertility, anti-depression, anti-microbial, pain relief, immunomodulatory activites.^98,99,100,101^ The active constituents (Figure 12) of moringa oleifera includes n-hexadecanoic acid **(73)**, tetradecanoic acid **(74)**, cis-vaccenic acid **(75)**, octadecanoic acid **(76)**, vitamin E **(77)**, gamma sitosterol **(78)**, squalane **(79)**, 2,6-dihydroxy benzoic acid **(80)**, quinic acid **(81)**, hexadecanal **(82)**, phytol **(83)**, panthonine **(84)**, vanillin **(85)**, moringine **(86)**, moringinine **(87).**^98,99^

*Moringa oleifera* has facilitated both humoral and cell mediated immune response by methanolic extract. The methanolic extract of *M. oleifera* shows effective immune response at low doses (250 mg/kg) when compared to high dose (750 mg/kg). From neutrophil adhesion assay, the methanolic extract of *M. oleifera* triggers neutrophil activation towards the site of inflammation. Diminishing the cyclophosphamide neutropenia at both low and high doses of methanolic extract indicates that cyclophosphamide’s effect on the haemopoietic system. The extract of *M. oleifera* at low and high doses show elevated levels of serum immunoglobulin. Polysaccharides isolated from the hot aqueous extract from *M. oleifera* mature pods demonstrated substantial macrophagic activity by releasing nitric oxide to the mouse cell line monocyte. An African study found that moringa powder supplementation could serve as an immune stimulant for HIV-infected patients.^102^ The extract of *M. oleifera* leaves show anti-oxidant activity against free radicals and prevent oxidative damage due to high amounts of polyphenols. *M. oleifera* can selectively inhibit the production of iNOS and COX-2 and also inhibits the secretion of nitric oxide and other inflammatory mediators like TNF-alpha, PGE-2, IL-6, IL-1beta in lipopolysaccharide induced RAW264.7 cells. Isothiocyanates extracted from the *M. oleifera* show a potent anti-cancer activity.^99^ Several studies of *M. oleifera* diet in chicken show better growth performance, anti-oxidant activity and better immune response.* M. oleifera* supplementation for boilers diets would modulate the immune responses by regulating the immune mediators like IL-2, IL-6.* M. oleifera* is given as a neutraceutical because of its safety to treat various chronic problems.^101^ After ingestion of moringa oleifera extract show reduced blood glucose levels within 3 hours.^100^

## Screening methods for immunomodulatory property

*In vitro* and animal models are used to test the effectiveness and toxicity of the active constituents, which are separated and extracted from a plant extract that illustrates the bioactivities. An overview of *in vitro* and *in vivo* models for assessment of immunomodulatory activity are summarized in Table 2.

**Table 2:** Screening methods for immunomodulation^12,103^

***In vitro *** **methods**	***In vivo*** ** methods**
Inhibition of histamine release from mast cells	Antianaphylactic activity (Schultz-Dale reaction)
Mitogen induced lymphocyte proliferation	Porcine cardiac myosin-induced autoimmune myocarditis in rats
Inhibition of T-cell proliferation	Acute systemic anaphylaxis in rats
Chemiluminescence in macrophages	Arthus-type immediate hypersensitivity
Plaque-forming colony test	Delayed type hypersensitivity
MTT assay	Passive cutaneous anaphylaxis
Neutrophil adhesion test	Adjuvant arthritis in rats
Hemagglutination antibody titer	Collagen type II induced arthritis in rats
Inducible nitric oxide synthase activity	Prevention of experimental induced myasthenia gravis in rats.
	Acute graft versus host disease in rats

## Conclusion

 Approximately 40% of medications, such as aspirin, anti-malarial, anti-cancer, digital, etc., have been marketed or used as a scaffold for the design of synthetic versions. At present only small fraction of plant studies have been performed to establish immunomodulatory activity. Therefore, the use of herbal remedies as a viable route for providing specific and wider range of immunomodulatory activity. Plant derived immunomodulators are an alternative therapy for conventional chemotherapy due to various side effects, high cost of synthetic compounds. Plant derived immunomodulators are prophylactic treatment and used for cure of various ailments and disorders offers a safer alternative. They are many natural supplements with potent pharmacological applications because they are remarkably efficient, affordable and low toxic. Natural products are the main constitutes as a lead molecule for designing and developing a new molecule for a therapeutic agent. Research on herbal medicines/products is enhanced around the world, as not only a part of traditional therapies but also for the management of health care. The WHO notes that the protection and efficiency of herbal usage remain a major concern. It would be clinically useful if genuine, standardised and quality regulated herbal products are tested if the principles of good practice on medicine are scientifically established in order to achieve results. Finally, the issue of how both individualization and herbal therapy are to be handled must be resolved. If these problems have been addressed, there is the possibility that herbal medicine will be commonly used as a healthcare that is healthy, reliable and accessible.

## Acknowledgments

 “We acknowledge the generous research infrastructure and supports from JSS College of Pharmacy, Ooty, the Nilgiris, Tamilnadu, India.”

## Ethical Issues

 Not Applicable.

## Conflict of Interest

 The authors report no conflict of interest.

## References

[1] Delves PJ, Martin SJ, Burton DR, Roitt IM. Roitt›s Essential Immunology. John Wiley & Sons; 2017.

[2] Kindt TJ, Goldsby RA, Osborne BA, Kuby J. Kuby Immunology. Macmillan; 2007.

[3] Quinn PJ (1990). Mechanisms of action of some immunomodulators used in veterinary medicine. Adv Vet Sci Comp Med.

[4] Albiger B, Dahlberg S, Henriques-Normark B, Normark S (2007). Role of the innate immune system in host defence against bacterial infections: focus on the Toll-like receptors. J Intern Med.

[5] Hansson GK, Libby P, Schönbeck U, Yan ZQ (2002). Innate and adaptive immunity in the pathogenesis of atherosclerosis. Circ Res.

[6] Slifka MK, Antia R, Whitmire JK, Ahmed R (1998). Humoral immunity due to long-lived plasma cells. Immunity.

[7] Behrens G, Li M, Smith CM, Belz GT, Mintern J, Carbone FR (2004). Helper T cells, dendritic cells and CTL Immunity. Immunol Cell Biol.

[8] Russell JH, Ley TJ (2002). Lymphocyte-mediated cytotoxicity. Annu Rev Immunol.

[9] Puri A, Saxena R, Saxena RP, Saxena KC, Srivastava V, Tandon JS (1994). Immunostimulant activity of Nyctanthes arbor-tristis L. J Ethnopharmacol.

[10] Song L, Xiong D, Hu M, Kang X, Pan Z, Jiao X (2016). Immunopotentiation of different adjuvants on humoral and cellular immune responses induced by HA1-2 subunit vaccines of H7N9 influenza in mice. PLoS One.

[11] Kumar D, Arya V, Kaur R, Bhat ZA, Gupta VK, Kumar V (2012). A review of immunomodulators in the Indian traditional health care system. J Microbiol Immunol Infect.

[12] Ahmad Khan MS, Ahmad I, Chattopadhyay D, eds. New Look to Phytomedicine: Advancements in Herbal Products as Novel Drug Leads. Academic Press; 2019. 10.1016/b978-0-12-814619-4.00018-5.

[13] Dahanukar SA, Thatte UM (1997). Current status of ayurveda in phytomedicine. Phytomedicine.

[14] Dorsch W, Stuppner H, Wagner H, Gropp M, Demoulin S, Ring J (1991). Antiasthmatic effects of Picrorhiza kurroa: androsin prevents allergen- and PAF-induced bronchial obstruction in guinea pigs. Int Arch Allergy Appl Immunol.

[15] Kotha RR, Luthria DL (2019). Curcumin: biological, pharmaceutical, nutraceutical, and analytical aspects. Molecules.

[16] Bhaumik S, Jyothi MD, Khar A (2000). Differential modulation of nitric oxide production by curcumin in host macrophages and NK cells. FEBS Lett.

[17] Catanzaro M, Corsini E, Rosini M, Racchi M, Lanni C (2018). Immunomodulators inspired by nature: a review on curcumin and Echinacea. Molecules.

[18] Momtazi-Borojeni AA, Mohammadian Haftcheshmeh S, Esmaeili SA, Johnston TP, Abdollahi E, Sahebkar A (2018). Curcumin: a natural modulator of immune cells in systemic lupus erythematosus. Autoimmun Rev.

[19] Singh S, Aggarwal BB (1995). Activation of transcription factor NF-kappa B is suppressed by curcumin (diferuloylmethane) [corrected]. J Biol Chem.

[20] Holden NS, Squires PE, Kaur M, Bland R, Jones CE, Newton R (2008). Phorbol ester-stimulated NF-kappaB-dependent transcription: roles for isoforms of novel protein kinase C. Cell Signal.

[21] Jantan I, Ahmad W, Bukhari SN (2015). Plant-derived immunomodulators: an insight on their preclinical evaluation and clinical trials. Front Plant Sci.

[22] Liu L, Liu YL, Liu GX, Chen X, Yang K, Yang YX (2013). Curcumin ameliorates dextran sulfate sodium-induced experimental colitis by blocking STAT3 signaling pathway. Int Immunopharmacol.

[23] Brück J, Holstein J, Glocova I, Seidel U, Geisel J, Kanno T (2017). Nutritional control of IL-23/Th17-mediated autoimmune disease through HO-1/STAT3 activation. Sci Rep.

[24] Chandrasekaran CV, Sundarajan K, Edwin JR, Gururaja GM, Mundkinajeddu D, Agarwal A (2013). Immune-stimulatory and anti-inflammatory activities of Curcuma longa extract and its polysaccharide fraction. Pharmacognosy Res.

[25] Sagrawat H, Khan MY (2007). Immunomodulatory plants: a phytopharmacological review. Pharmacogn Rev.

[26] Rajagopal S, Kumar RA, Deevi DS, Satyanarayana C, Rajagopalan R (2003). Andrographolide, a potential cancer therapeutic agent isolated from Andrographis paniculata. J Exp Ther Oncol.

[27] Jayakumar T, Hsieh CY, Lee JJ, Sheu JR (2013). Experimental and clinical pharmacology of Andrographis paniculata and its major bioactive phytoconstituent andrographolide. Evid Based Complement Alternat Med.

[28] Lee KC, Chang HH, Chung YH, Lee TY (2011). Andrographolide acts as an anti-inflammatory agent in LPS-stimulated RAW2647 macrophages by inhibiting STAT3-mediated suppression of the NF-κB pathway. J Ethnopharmacol.

[29] Zhang QQ, Ding Y, Lei Y, Qi CL, He XD, Lan T (2014). Andrographolide suppress tumor growth by inhibiting TLR4/NF-κB signaling activation in insulinoma. Int J Biol Sci.

[30] Nanduri S, Nyavanandi VK, Thunuguntla SS, Kasu S, Pallerla MK, Ram PS (2004). Synthesis and structure-activity relationships of andrographolide analogues as novel cytotoxic agents. Bioorg Med Chem Lett.

[31] Matsuda T, Kuroyanagi M, Sugiyama S, Umehara K, Ueno A, Nishi K (1994). Cell differentiation-inducing diterpenes from Andrographis paniculata Nees. Chem Pharm Bull (Tokyo).

[32] Calabrese C, Berman SH, Babish JG, Ma X, Shinto L, Dorr M (2000). A phase I trial of andrographolide in HIV positive patients and normal volunteers. Phytother Res.

[33] Uttekar MM, Das T, Pawar RS, Bhandari B, Menon V, Nutan Nutan (2012). Anti-HIV activity of semisynthetic derivatives of andrographolide and computational study of HIV-1 gp120 protein binding. Eur J Med Chem.

[34] Akowuah GA, Zhari I, Mariam A, Yam MF (2009). Absorption of andrographolides from Andrographis paniculata and its effect on CCl4-induced oxidative stress in rats. Food Chem Toxicol.

[35] Verma N, Vinayak M (2008). Antioxidant action of Andrographis paniculata on lymphoma. Mol Biol Rep.

[36] Zhang XF, Tan BK (2000). Anti-diabetic property of ethanolic extract of Andrographis paniculata in streptozotocin-diabetic rats. Acta Pharmacol Sin.

[37] Melchior J, Palm S, Wikman G (1997). Controlled clinical study of standardized Andrographis paniculata extract in common cold-a pilot trial. Phytomedicine.

[38] Iruretagoyena MI, Tobar JA, González PA, Sepúlveda SE, Figueroa CA, Burgos RA (2005). Andrographolide interferes with T cell activation and reduces experimental autoimmune encephalomyelitis in the mouse. J Pharmacol Exp Ther.

[39] Burgos RA, Caballero EE, Sánchez NS, Schroeder RA, Wikman GK, Hancke JL (1997). Testicular toxicity assessment of Andrographis paniculata dried extract in rats. J Ethnopharmacol.

[40] Qin LH, Kong L, Shi GJ, Wang ZT, Ge BX (2006). Andrographolide inhibits the production of TNF-alpha and interleukin-12 in lipopolysaccharide-stimulated macrophages: role of mitogen-activated protein kinases. Biol Pharm Bull.

[41] Percival SS (2000). Use of Echinacea in medicine. Biochem Pharmacol.

[42] Barrett B (2003). Medicinal properties of Echinacea: a critical review. Phytomedicine.

[43] Chicca A, Adinolfi B, Martinotti E, Fogli S, Breschi MC, Pellati F (2007). Cytotoxic effects of Echinacea root hexanic extracts on human cancer cell lines. J Ethnopharmacol.

[44] Luettig B, Steinmüller C, Gifford GE, Wagner H, Lohmann-Matthes ML (1989). Macrophage activation by the polysaccharide arabinogalactan isolated from plant cell cultures of Echinacea purpurea. J Natl Cancer Inst.

[45] Fonseca FN, Papanicolaou G, Lin H, Lau CB, Kennelly EJ, Cassileth BR (2014). Echinacea purpurea (L) Moench modulates human T-cell cytokine response. Int Immunopharmacol.

[46] See DM, Broumand N, Sahl L, Tilles JG (1997). In vitro effects of Echinacea and ginseng on natural killer and antibody-dependent cell cytotoxicity in healthy subjects and chronic fatigue syndrome or acquired immunodeficiency syndrome patients. Immunopharmacology.

[47] Steinmüller C, Roesler J, Gröttrup E, Franke G, Wagner H, Lohmann-Matthes ML (1993). Polysaccharides isolated from plant cell cultures of Echinacea purpurea enhance the resistance of immunosuppressed mice against systemic infections with Candida albicans and Listeria monocytogenes. Int J Immunopharmacol.

[48] Rondanelli M, Miccono A, Lamburghini S, Avanzato I, Riva A, Allegrini P (2018). Self-care for common colds: the pivotal role of vitamin D, vitamin C, Zinc, and Echinacea in three main immune interactive clusters (physical barriers, innate and adaptive immunity) involved during an episode of common colds-practical advice on dosages and on the time to take these nutrients/botanicals in order to prevent or treat common colds. Evid Based Complement Alternat Med.

[49] Sultan MT, Butt MS, Qayyum MM, Suleria HA (2014). Immunity: plants as effective mediators. Crit Rev Food Sci Nutr.

[50] Saha S, Ghosh S (2012). Tinospora cordifolia: one plant, many roles. Anc Sci Life.

[51] Singh N, Singh SM, Shrivastava P (2004). Immunomodulatory and antitumor actions of medicinal plant Tinospora cordifolia are mediated through activation of tumor-associated macrophages. Immunopharmacol Immunotoxicol.

[52] Rege NN, Thatte UM, Dahanukar SA (1999). Adaptogenic properties of six rasayana herbs used in Ayurvedic medicine. Phytother Res.

[53] Kapil A, Shah R, Sharma S (1994). Immunopotentiating and anticomplementary activity of fungisterol from mushroom Suillus sibiricus. Indian Drugs.

[54] More P, Pai K (2012). In vitro NADH-oxidase, NADPH-oxidase and myeloperoxidase activity of macrophages after Tinospora cordifolia (guduchi) treatment. Immunopharmacol Immunotoxicol.

[55] Mathew S, Kuttan G (1999). Immunomodulatory and antitumour activities of Tinospora cordifolia. Fitoterapia.

[56] Alzohairy MA (2016). Therapeutics role of Azadirachta indica (Neem) and their active constituents in diseases prevention and treatment. Evid Based Complement Alternat Med.

[57] Girish K, Shankara BS (2008). Neem–a green treasure. Electron J Biol.

[58] Hwisa NT, Assaleh FH, Gindi S, Melad FE, Chandu BR, Katakam P (2014). A study on antiurolithiatic activity of Melia azadirachta L aqueous extract in rats. Am J Pharmacol Sci.

[59] Hossain MA, Shah MD, Sakari M (2011). Gas chromatography-mass spectrometry analysis of various organic extracts of Merremia borneensis from Sabah. Asian Pac J Trop Med.

[60] Kumar S, Suresh PK, Vijayababu MR, Arunkumar A, Arunakaran J (2006). Anticancer effects of ethanolic neem leaf extract on prostate cancer cell line (PC-3). J Ethnopharmacol.

[61] Upadhyay SN, Dhawan S, Garg S, Talwar GP (1992). Immunomodulatory effects of neem (Azadirachta indica) oil. Int J Immunopharmacol.

[62] Talwar GP, Raghuvanshi P, Misra R, Mukherjee S, Shah S (1997). Plant immunomodulators for termination of unwanted pregnancy and for contraception and reproductive health. Immunol Cell Biol.

[63] SaiRam M, Sharma SK, Ilavazhagan G, Kumar D, Selvamurthy W (1997). Immunomodulatory effects of NIM-76, a volatile fraction from Neem oil. J Ethnopharmacol.

[64] Sadekar RD, Kolte AY, Barmase BS, Desai VF (1998). Immunopotentiating effects of Azadirachta indica (Neem) dry leaves powder in broilers, naturally infected with IBD virus. Indian J Exp Biol.

[65] Moutia M, Habti N, Badou A (2018). In vitro and in vivo immunomodulator activities of Allium sativum L. Evid Based Complement Alternat Med.

[66] Arreola R, Quintero-Fabián S, López-Roa RI, Flores-Gutiérrez EO, Reyes-Grajeda JP, Carrera-Quintanar L (2015). Immunomodulation and anti-inflammatory effects of garlic compounds. J Immunol Res.

[67] Harper JM (1978). Food extrusion. CRC Crit Rev Food Sci Nutr.

[68] Harris JC, Cottrell SL, Plummer S, Lloyd D (2001). Antimicrobial properties of Allium sativum (garlic). Appl Microbiol Biotechnol.

[69] Mohamed HZE, Shenouda MBK (2021). Amelioration of renal cortex histological alterations by aqueous garlic extract in gentamicin induced renal toxicity in albino rats: a histological and immunohistochemical study. Alexandria J Med.

[70] Quintero-Fabián S, Ortuño-Sahagún D, Vázquez-Carrera M, López-Roa RI (2013). Alliin, a garlic (Allium sativum) compound, prevents LPS-induced inflammation in 3T3-L1 adipocytes. Mediators Inflamm.

[71] Kim SR, Jung YR, An HJ, Kim DH, Jang EJ, Choi YJ (2013). Anti-wrinkle and anti-inflammatory effects of active garlic components and the inhibition of MMPs via NF-κB signaling. PLoS One.

[72] Feng Y, Zhu X, Wang Q, Jiang Y, Shang H, Cui L (2012). Allicin enhances host pro-inflammatory immune responses and protects against acute murine malaria infection. Malar J.

[73] Mishra S, Aeri V, Gaur PK, Jachak SM (2014). Phytochemical, therapeutic, and ethnopharmacological overview for a traditionally important herb: Boerhavia diffusa Linn. Biomed Res Int.

[74] Selvaraj D, Shanmughanandhan D, Sarma RK, Joseph JC, Srinivasan RV, Ramalingam S (2012). DNA barcode ITS effectively distinguishes the medicinal plant Boerhavia diffusa from its adulterants. Genomics Proteomics Bioinformatics.

[75] Bhowmik D, Kumar KS, Paswan S, Srivatava S, Yadav A, Dutta A (2012). Traditional Indian herbs Convolvulus pluricaulis and its medicinal importance. J Pharmacogn Phytochem.

[76] Mehrotra S, Mishra KP, Maurya R, Srimal RC, Singh VK (2002). Immunomodulation by ethanolic extract of Boerhaavia diffusa roots. Int Immunopharmacol.

[77] Manu KA, Kuttan G (2007). Effect of Punarnavine, an alkaloid from Boerhaavia diffusa, on cell-mediated immune responses and TIMP-1 in B16F-10 metastatic melanoma-bearing mice. Immunopharmacol Immunotoxicol.

[78] Sumanth M, Mustafa SS (2007). Antistress, adoptogenic and immunopotentiating activity roots of Boerhaavia diffusa in mice. Int J Pharmacol.

[79] Stohs SJ, Bagchi D (2015). Antioxidant, anti-inflammatory, and chemoprotective properties of Acacia catechu heartwood extracts. Phytother Res.

[80] Li X, Wang H, Liu C, Chen R (2010). [Chemical constituents of Acacia catechu]. Zhongguo Zhong Yao Za Zhi.

[81] Li XC, Liu C, Yang LX, Chen RY (2011). Phenolic compounds from the aqueous extract of Acacia catechu. J Asian Nat Prod Res.

[82] Guleria S, Tiku AK, Singh G, Vyas D, Bhardwaj A (2011). Antioxidant activity and protective effect against plasmid DNA strand scission of leaf, bark, and heartwood extracts from Acacia catechu. J Food Sci.

[83] Ghate NB, Hazra B, Sarkar R, Mandal N (2014). Heartwood extract of Acacia catechu induces apoptosis in human breast carcinoma by altering Bax/Bcl-2 ratio. Pharmacogn Mag.

[84] Ismail S, Asad M (2009). Immunomodulatory activity of Acacia catechu. Indian J Physiol Pharmacol.

[85] Kang S, Min H (2012). Ginseng, the ‹immunity boost›: the effects of Panax ginseng on immune system. J Ginseng Res.

[86] Shin JY, Song JY, Yun YS, Yang HO, Rhee DK, Pyo S (2002). Immunostimulating effects of acidic polysaccharides extract of Panax ginseng on macrophage function. Immunopharmacol Immunotoxicol.

[87] Lim DS, Bae KG, Jung IS, Kim CH, Yun YS, Song JY (2002). Anti-septicaemic effect of polysaccharide from Panax ginseng by macrophage activation. J Infect.

[88] Wang H, Actor JK, Indrigo J, Olsen M, Dasgupta A (2003). Asian and Siberian ginseng as a potential modulator of immune function: an in vitro cytokine study using mouse macrophages. Clin Chim Acta.

[89] Kim MH, Byon YY, Ko EJ, Song JY, Yun YS, Shin T (2009). Immunomodulatory activity of ginsan, a polysaccharide of Panax ginseng, on dendritic cells. Korean J Physiol Pharmacol.

[90] Takei M, Tachikawa E, Hasegawa H, Lee JJ (2004). Dendritic cells maturation promoted by M1 and M4, end products of steroidal ginseng saponins metabolized in digestive tracts, drive a potent Th1 polarization. Biochem Pharmacol.

[91] Su W, Sun AJ, Xu DL, Zhang HQ, Yang L, Yuan LY (2010). Inhibiting effects of total saponins of Panax ginseng on immune maturation of dendritic cells induced by oxidized-low density lipoprotein. Cell Immunol.

[92] Tung NH, Quang TH, Son JH, Koo JE, Hong HJ, Koh YS (2011). Inhibitory effect of ginsenosides from steamed ginseng-leaves and flowers on the LPS-stimulated IL-12 production in bone marrow-derived dendritic cells. Arch Pharm Res.

[93] Liou CJ, Huang WC, Tseng J (2006). Short-term oral administration of ginseng extract induces type-1 cytokine production. Immunopharmacol Immunotoxicol.

[94] Yoo DG, Kim MC, Park MK, Park KM, Quan FS, Song JM (2012). Protective effect of ginseng polysaccharides on influenza viral infection. PLoS One.

[95] Kim HA, Kim S, Chang SH, Hwang HJ, Choi YN (2007). Anti-arthritic effect of ginsenoside Rb1 on collagen induced arthritis in mice. Int Immunopharmacol.

[96] O›Hagan DT, Valiante NM (2003). Recent advances in the discovery and delivery of vaccine adjuvants. Nat Rev Drug Discov.

[97] Song X, Chen J, Sakwiwatkul K, Li R, Hu S (2010). Enhancement of immune responses to influenza vaccine (H3N2) by ginsenoside Re. Int Immunopharmacol.

[98] Kou X, Li B, Olayanju JB, Drake JM, Chen N (2018). Nutraceutical or pharmacological potential of Moringa oleifera Lam. Nutrients.

[99] Bhattacharya A, Tiwari P, Sahu PK, Kumar S (2018). A review of the phytochemical and pharmacological characteristics of Moringa oleifera. J Pharm Bioallied Sci.

[100] Dubey DK, Dora J, Kumar A, Gulsan RK (2013). A multipurpose tree-Moringa oleifera. Int J Pharm Chem Sci.

[101] Jacques AS, Arnaud SS, Fréjus OO, Jacques DT (2020). Review on biological and immunomodulatory properties of Moringa oleifera in animal and human nutrition. J Pharmacogn Phytother.

[102] Sudha P, Asdaq SM, Dhamingi SS, Chandrakala GK (2010). Immunomodulatory activity of methanolic leaf extract of Moringa oleifera in animals. Indian J Physiol Pharmacol.

[103] Singh N, Tailang M, Mehta SC (2016). A review on herbal plants as immunomodulators. Int J Pharm Sci Res.

